# Sero-prevalence of hepatitis viral infections among sanitary workers across worldwide: a systematic review and meta-analysis

**DOI:** 10.1186/s12879-023-08354-1

**Published:** 2023-06-13

**Authors:** Sina Tolera, Dechasa Adare Mengistu, Fekade Ketema Alemu, Abraham Geremew, Yohannes Mulugeta, Gebisa Dirirsa, Liku Muche Temesgen, Wegene Diriba, Gutema Mulatu, Tamagnu Sintie, Kefelegn Bayu, Ashenafi Berhanu

**Affiliations:** School of Environmental Health, College of Health and Medical Sciences, Haramaya University, P.O.Box:235, Harar, Ethiopia

**Keywords:** Hepatitis Virus, Infections, Occupation, Sanitary workers, Worldwide

## Abstract

**Background:**

Sanitation or sanitary workers are exposed to hepatitis virus infections because of filthy and dangerous working conditions. The current global systematic review and meta-analysis aimed to estimate the pooled sero-prevalence of occupationally associated hepatitis virus infection among them.

**Methods:**

Preferred Reporting Items for Systematic Reviews (PRISMA), and Population, Intervention, Comparison, Outcome and study design (PICOS) were used for flow diagram, and review questions, respectively. Four databases other methods were used published articles from 2000 to 2022. Boolean logic (AND, OR), MeSH, and keywords were used: (Occupation *OR Job *OR Work) AND (Hepatitis A *OR Hepatitis B virus *OR Hepatitis C virus *OR Hepatitis E virus) AND (Solid waste collectors [SWCs] *OR Street sweepers [SS] *OR Sewage workers [STWs] *OR health care facilities cleaners [HCFCs)) AND (Countries). Stata MP/17 software was used for pooled prevalence analysis, meta-regression analysis (Hedges) at a 95% confidence interval (CI:95%).

**Results:**

A total of 182 studies were identified studies, a total of 28 studies were included from twelve countries. Of these, from developed (*n *= 7) and developing countries (*n* = 5). From total a of 9049 sanitary workers, 5951(66%), 2280 (25%) and 818 (9%) were STWs, SWCs and SS, respectively. Globally, the pooled sero-prevalence of occupational-related hepatitis viral infections among sanitary workers was 38.06% (95% CI: 30–0.46.12). Of this, it was 42.96% (95% CI: 32.63–53.29) and 29.81% (95% CI: 17.59–42.02) for high-income and low-income countries, respectively. Meanwhile, by sub-analysis, the highest pooled sero-prevalence of hepatitis viral infections by categories, type and year were 47.66% (95%CI: 37.42–57.90), 48.45% (95% CI: 37.95–58.96), and 48.30% (95% CI: 36.13–60.47) for SWTs, HAV, and 2000 to 2010 year, respectively.

**Conclusion:**

The consistency of the evidence suggests that sanitation workers, particularly sewage workers, are susceptible to occupationally acquired hepatitis regardless of their working conditions, necessitating significant changes to occupational health and safety regulations from governmental policies and other initiatives to reduce risks among sanitary workers.

**Supplementary Information:**

The online version contains supplementary material available at 10.1186/s12879-023-08354-1.

## Introduction

Workers in sanitation are crucial to public health and societal well-being all around the world [1–3]; and they are maintaining safe sanitation services in homes, schools, hospitals, and other settings and protecting public health [4]. However, due to poor occupational health and safety practice, a numerous studies reported from sanitary workers are the possibilities of exposing with excreted bodily fluids, blood with infectious waste is material suspected to contain pathogens (such as bacteria, viruses, parasites or fungi) [5–7]. As numerous studies indicated sewage workers and waste treatment workers, solid waste collectors, street sweepers and health care cleaners the possibility of develop hepatitis viral infections (such as hepatitis A virus, hepatitis B virus, hepatitis C virus, hepatitis E virus and other occupational related diseases) [8]. Of these viral infections, hepatitis A virus (HAV) infection is common, in which the transmission of the disease occurs by the faecal-oral route. The other viral is hepatitis B virus infection, in which transmitted through perinatal, sexual and parenteral/percutaneous at elsewhere [9], which is one of the most common infectious diseases globally. Hepatitis B virus (HBV) is likely to be more prevalent in occupational groups in solid waste collectors [10].

As the study reported, sanitary workers are at a high risk of a variety of injuries and infections, such as HIV and hepatitis through exposure to infected needles/sharp objects in wastes, which may lead to disease transmission [11]. As the worldwide, 350 million chronic hepatitis B virus (HBV) carriers were estimated among these groups.. The prevalence of chronic HBV infection varies geographically, from high (> 8%), intermediate (2–7%) to low (< 2%) prevalence. HBeAg-negative chronic hepatitis B (e-CHB) and occult HBV infection are two special clinical entities, and the prevalence and clinical implications remain to be explored [12].

The third one is hepatitis virus C that occurred after percutaneous exposure, the majority from hollow-bore needles disposed in the waste used in the source patient's vein or artery and contaminated with blood or blood-stained fluid [13]. The other one is hepatitis E virus (HEV) infection that is endemic in many developing countries, causing substantial morbidity. transmission is primarily faeco-oral and is associated with both sporadic infections and epidemics in areas where poor sanitation and weak public health infrastructures exist [14]. Furthermore, sanitary workers who handle human waste or sewage may be more susceptible to waterborne infections. Use basic methods connected with wastewater treatment plant operations to limit this risk and safeguard against illnesses such as diarrhea. Engineering and administrative controls, sanitary measures, particular safe work practices, and personal protective equipment (PPE) are all examples of standard procedures [15]. Therefore, the major purpose of this systematic review and meta-analysis was to estimate pool sero-prevalence of hepatitis virus infections related with vocations worldwide, in low-to-high income nations, and in a sub-group that is neither widely known nor fully recorded.

## Method and materials

### Review protocols

Preferred Reporting Items for Systematic Reviews (PRISMA) updated criteria [16] protocol was used flow diagram of the articles. Meanwhile, the PICOS *(* Population [P], Intervention[I]*,* Comparison[C], Outcome[O], and Study type[S] protocol was used for formulated question and desire search strategies.

### Databases and searched strategies

ST, DA, FA, and AG contributed by searching for published articles online with EndNote. Data were searched from PubMed, Google Scholar, MEDLINE, CINAHL, Science Direct, Web of Science, and the Directory of Open Access Journals, as well as catalogs, homepages, and reports. The keywords and MeSH terms with Boolean logic (AND, OR) for searching strategies was (Occupational *OR Job *OR Work Associated *OR Related) AND (Hepatitis Virus Infections [A, B, C, E]) AND (Street Sweepers *OR Solid waste collectors *OR Municipality Solid Waste Collectors *OR Solid Waste Collectors *OR Garbage workers *OR Sewage workers *OR Waste Treatment worker *OR Health Care Facility Cleaners) AND Countries (High-income countries *OR Industrial countries *OR Developed countries AND/OR Low-income countries *OR Poor nations *OR Developing countries).

### Eligibility criteria

The PICOS protocol (Population, Intervention, Comparison, Outcome, Study Design) was utilized for eligibility criteria, which are detailed below.

#### Inclusion criteria

i) *Population*: Stands for Sanitary workers namely solid waste collectors, health care facility cleaners, sewage workers and waste water treatment workers and sweeping streets;
ii) *Intervention:* Occupational exposure
iii) *Comparison:* Not applicable
iv) *Outcome:* Occupationally associated or occupational related prevalence of hepatitis viral infections namely hepatitis A virus, hepatitis B virus, hepatitis C virus and hepatitis E virus were included
v) *Study type:* Cross Sectional study design
vi) *Language:* All Articles/studies published in English Language were included
vii) *Articles/Studies:* This review covered articles with complete texts and abstracts available as well as clear objectives and methods, studies included, and quantitative outcomes.
viii) *Publication:* Only published articles between 2000 and 2022 years were included.

#### Exclusion criteria

i) *Population:* Office cleaners, Hotel and Restaurant cleaners were excluded in this review due to their work type and characteristic their job.
ii) *Study Design:* Non-cross-sectional studies like randomized controlled trials that are individually-or cluster- randomized controlled trials. he following non-randomized controlled studies: quasi- randomized controlled trials, non- randomized controlled trials, historically controlled studies, time-series studies, case–control and cohort studies.
iii) *Outcomes:* Studies conducted on occupational related injuries, musculoskeletal disorders, mental health conditions; occupationnaly associated fungi, bacteria parasites and other viral infection were excluded
iv) *Articles/Studies:* Full or abstract articles/studies with no defined purpose or methodology were excluded.
v) *Language:* Studies published with non-English languages were excluded
vi) *Publication:* Studies prior to 2000 years didn’t include in this review*.*

### Data screening

YM, LM, GM, GD, AB and WD searched and screened article using Microsoft Excel and full copies of titles and abstracts were obtained. Then finally, the results from the databases were managed and removed in the reference management EndNote 20.4.1 and Zotero program respectively.

### Data extraction

ST, FA, TS, KB, and AB extracted data from a Microsoft Excel file using a predefined extraction form. The main author, year, reference number, country, study design, sanitary worker job categories, instrument used for outcomes assessment (prevalence of hepatitis infections asseessmnt tools), outcomes type ( HAV,HBV,HCV and HEV), and quality evaluation are all included.

### Data analysis

ST, DA and FA contributed on data analysis using Stata version MP/17. The analysis was done based on 28 studies. The effect size index was the event rate (Prevalence). Forest plot random-effect model (Restricted maximum–likelihood) was used to estimate the pooled sero-prevalence, sub-analysis pooled prevalence for by countries, by types of hepatitis infections, and by year with the confidence interval of 95%. Moreover, meta-regression (Random effect using Hedges method) was used test heterogeinity of eligibled studies. Here, I-square ( I^2^) test was used to examine the reported prevalence for heterogeneity. Sensitivity analysis was done after removing equal of prevalence of hepatitis vrial infections for the smallest (*n* = 3) and largest (*n* = 3) at *p*-value of 0.05(CI:95%). In addition, Virsual funnel plot was used to detect the publication bias at *p*-value 0.05 (CI:95%).

### Data synthesis

ST, YM, LM, TS, KB, GD, GM and DA contributed to this work through data synthesis, and data description and compilation from the exctrated teams based on the characteristics of the original articles using texts, tables and figures.

### Publication bias

Quality of the articles was evaluated by ST, DA, AG, YM, WD, AB and FA using the Joanna Briggs Institute (JBI) Critical appraisal checklist, which comprised 28 papers, was evaluated based on JBI standards and nine claims for cross-sectional studies adapted from [17]. All criteria measured as 1) Yes, 2) No 3) Unclear; 4) Not applicable. Finally, if the article received less than five points out of nine "Yes," it indicates a high publication risk or low paper quality, 5–7 indicates a medium publication risk, and 8–9 indicates a low publication bias. Moreover, using visual funnel plot was used to assess the possibility of publication bias and *p* value of 0.05 was considered evidence of publication bias.

## Result

### Selection studies

**Fig. 1:**
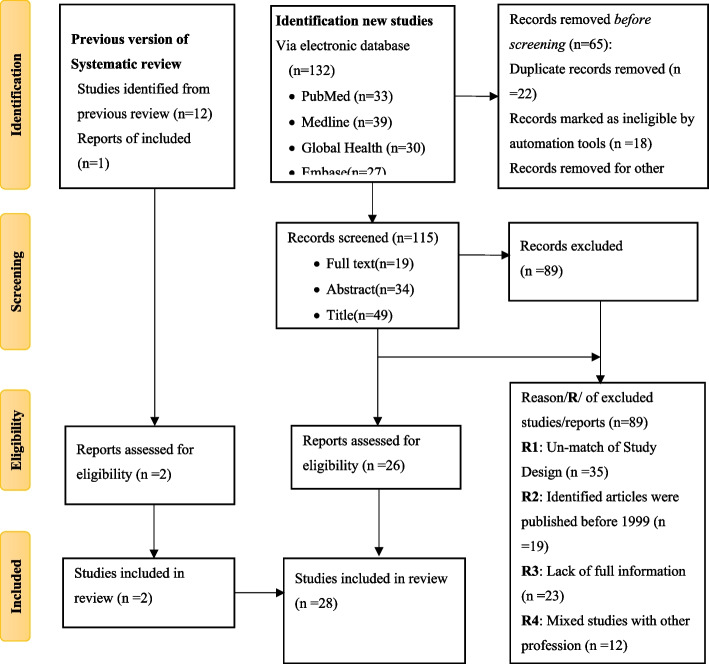
Screening Process. Flow diagram for systematic review and Meta-Analysis is adapted from PRISMA 2020 Protocol

A total of 182 studies were identified from the databases and other retrieved data and reports. Of these, 12 studies were from studies included in the previous systematic review, 129 studies were from new studies via databases, and 38 studies from new studies via others methods. Finally, 28 papers were included in order to determine occupationally associated sero-prevalence among sanitary workers (Fig. 1).

### Study overview

Twenty-eight studies were eligible studies that presented as authors, countries, study design, tool used, categories and number of sanitary workers, outcomes (type and prevamece of hepatitis viral infections) and publication bias (Table 1).

**Table 1:** Eligible studies included in review with Authors, Publication Year, Countries, Design, studied Populations, Outcomes and publication bias

**Authors**	**Pub. Year**	**Country**	**Design**	**Tool used**	**Study Pop. (*N* = **8,618**)**	Prevalence of Hepatitis	**Publ. bias**	**Ref**
Rachiotis et al	2012a	Greece	CS	Questionnaires	SWCs (*n* = 100)	Overall (61%) of HAV infection	Low	[18]
Rachiotis et al	2016	Greece	CS	Questionnaires	SWCs (*n* = 133)	Overall (50.7%) of HAV infection	Low	[19]
Arvanitidou et al	2004	Greece	CS	Questionnaires	Sewage (*n* = 108)	Overall (40%) of HAV infection	Medium	[20A]
Arvanitidou et al	2004	Greece	CS	Questionnaires	Sewage (*n* = 108)	Overall (65.7%) of HAV infection	Medium	[20B]
Rachiotis et al	2012b	Greece	CS	Questionnaires	SWCs (*n* = 208)	Overall (37.9%) of HAV infection	Low	[21]
Moraitaki et al	2010	Greece	CS	HAV IgG detection	SS(*n* = 49)	Overall (91.3%) of HAV infection	High	[22]
Bonanni et al	2000	Italy	CS	Serological analysis	Sewage (*n* = 225)	Overall (82%) of HAV infection	Medium	[23]
Divizia et al	2008	Italy	CS	questionnaire, blood	Sewage (*n* = 138)	Overall (52.7%) of HAV infection	Low	[24]
Montuori et al	2009	Italy	CS	Blood serology	WTW (*n* = 869)	Overall (38%) of HAV infection	Medium	[25]
Levin et al	2000	Israel	CS	Serological analysis	Sewage (*n* = 100)	Overall (67%) of HAV infection	Low	[26]
Toseva et al	2008	Bulgaria	CS	Blood sample	Wastewater (*n* = 110)	Overall (20%) of HAV infection	Medium	[27]
Vencze et al	2003	USA	CS	Blood sample	Sewage (*n* = 365)	Overall (50%) of HAV infection	Medium	[28]
Trout et al	2000	USA City	CS	Serology analysis (Blood)	Sewage (*n* = 163)	Overall (80.7%) of HAV infection	Medium	[29]
Weldon et al	2000	Texas	CS	Serology analysis (Blood)	Sewage (*n* = 359)	Overall (23%) of HBV infection	Medium	[30]
Benbrik et al	2000	Brazil	CS	Blood Sample	Sewage (*n* = 591)	Overall (32.4%) of HBV infection	Medium	[31]
Mariho et al	2014	Brazil	CS	Blood Sample	SWCs (*n* = 431)	Overall (36.1%) of HBV infection	Low	[32]
Ariyarathna & Abeysena	2019	Sri Lanka	CS	Blood Sample	Sewage (*n* = 1403)	Overall (12.8%) of HBV infection	Low	[33]
El-Wahab et al	2015	Egypt	CS	Questionnaires	SWCs/(*n* = 346)	Overall (12.2%) of HCV infection	Medium	[34]
Hassanein et al	2019	Egypt	CS	Blood and Stool sample	Sewage (*n* = 410)	Overall (10%) of HCV infection	Low	[35]
El-Gilany et al	2013	Egypt	CS	Blood sample	SWC(*n* = 120)	Overall (43.3%) of HCV infection	Low	[36]
Elkhateeb et al	2019	Egypt	CS	Blood sample	SWC(*n* = 171)	Overall (21.6%) of HCV infection	Medium	[37]
El-Esnawy	2000	Egypt	CS	HEV IgG detection	Sewage (*n* = 205)	Overall (56.5%) of HEV infection	Medium	[38]
Wanjari & Mendhe	2021	India	CS	Blood sample	SWC(*n* = 100)	Overall (16.1%) of HEV infection	Low	[39]
Vaidya et al	2003	India	CS	Blood sample	SW(*n* = 147)	Overall (19.23%) of HEV infection	Low	[40]
Hosseini et al	2022	Iran	CS	Blood sample	SS(*n* = 385)	Overall (50.65%) of HEV infectious	Medium	[41]
Farooqi et al	2022	Pakistan	CS	Blood Sample	Sewage exp (*n* = 650)	Overall (6.6%) due to HBV (8.3%) and HCV infectious (5%)	Medium	[42]
Raufu et al	2022	Nigeria	CS	Blood Sample	SWCs(*n* = 240)	Overall (32.6%) due to HAV	Medium	[43]
Erfani et al	2020	Iran	CS	Blood Sample	SS(*n* = 384)	Overall (26.3%) due to HAV	Medium	[44]

*Ref* Reference Number, *CS* Cross Sectional Study, *SCWs* Solid Waste Collectors; *SW* Sewage workers, *WTWs*: Waste treatment workers, Sewage *exp.* Sewage exposures, *SS* Street Sweepers

### Eligible countries

A total of twelve countries were eligible for current pooled seroprevalence of hepatitis viral infections among sanitary workers. Of these developed countries (*n* = 7) and developing countries (*n* = 5) (Sup Table 1). Majority of the studies found from Greece (*n* = 6) and Egypt (*n* = 5). From Italy (*n* = 3) and USA (*n* = 3) studies were identified (Sup Table 1).

### Eligible population

From total population of 9,049 sanitary workers, 5951 (66%) of them worked in sewage and waste treatment or liquid waste treatment. The second category included 2280 (25%) solid waste collectors and 818 (9%) of them were worked as street sweepers (Sup. Figure 1).

### Design and assessment tools

From included papers, those used cross-sectional studies with blood samples (serology analysis, were 19 studies (*n* = 19). Then followed by standard questionnaires alone (*n* = 6), machine detectors (*n* = 2), and questionnaires with serology analysis (*n* = 1) were used to assess the occupationally associated hepatitis viral infections (Sup. Figure 2).

### Sero-prevalence of hepatitis as globe and by regions

**Fig. 2:**
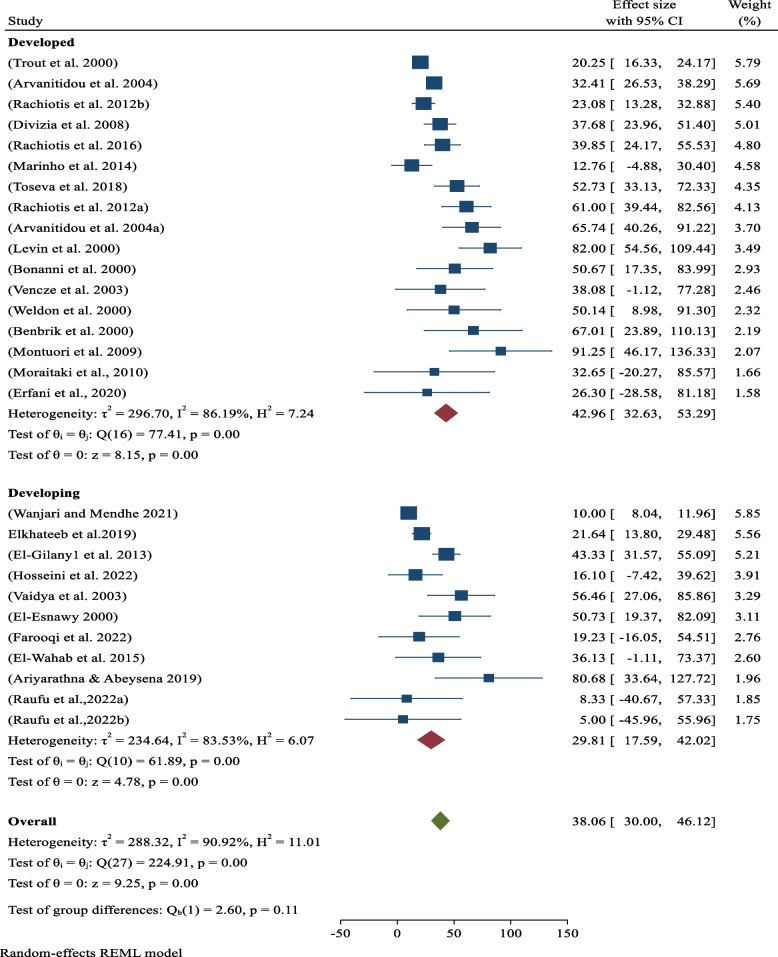
Analysis by Globe and Regions. Sero-prevalence of occupational-related hepatitis among sanitary workers by regions

Across the globe, the cumulative sero-prevalence of occupational-related all hepatitis infections among sanitary workers was 38.06% (95% CI: 30–0.46.12; *p*-value < 0.05) which was statisitically significant with their work condition. Of these, was 42.96% (95% CI: 32.63–53.29; *p*-value < 0.05) for high-income countries and 29.81% (95% CI: 17.59–42.02; *p*-value < 0.05) for low-income countries(Fig. 2).

### Sero-prevalence of hepatitis by occupations

**Fig. 3:**
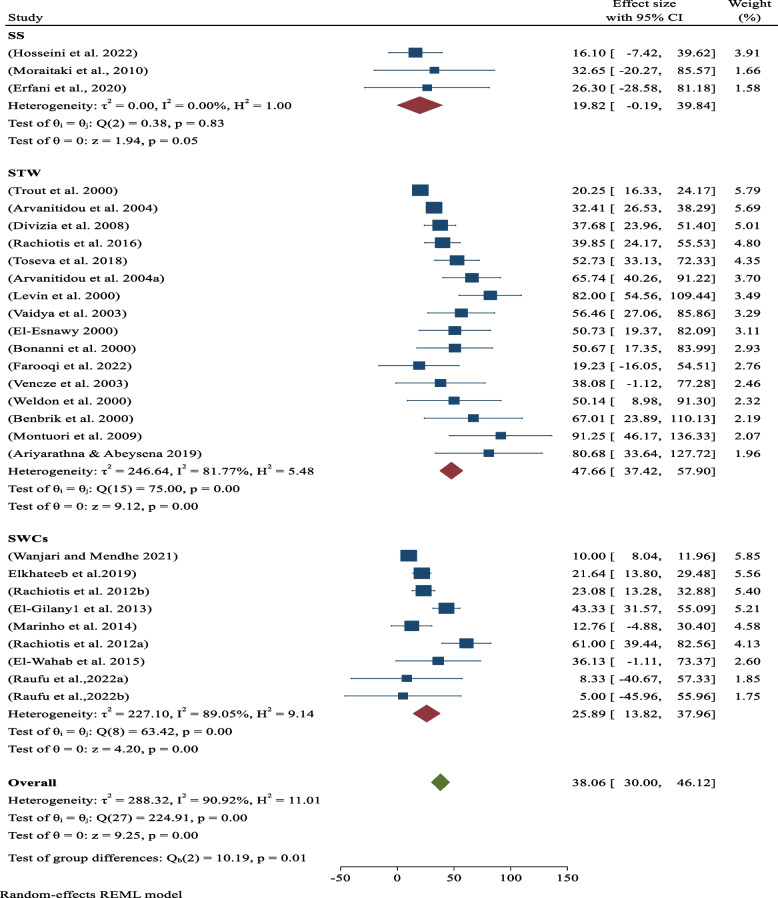
Analysis by Occupations (Categories). Sero-prevalence of hepatitis by occupations within subgroup of sanitary workers

By sub-group analysis, the pooled sero-prevalence of hepatitis viral infections among sewage and waste treatment was 47.66% (95%CI: 37.42–57.90; *p*-value < 0.05). It was 25.89% (95%CI: 13.82–37.96; *p*-value < 0.05) and 19.82% (95%CI: -0.19–39.84; *p*-value < 0.05) for solid waste collectors and street sweepers, respectively (Fig. 3).

### Sero-prevalence of hepatitis by type

**Fig. 4:**
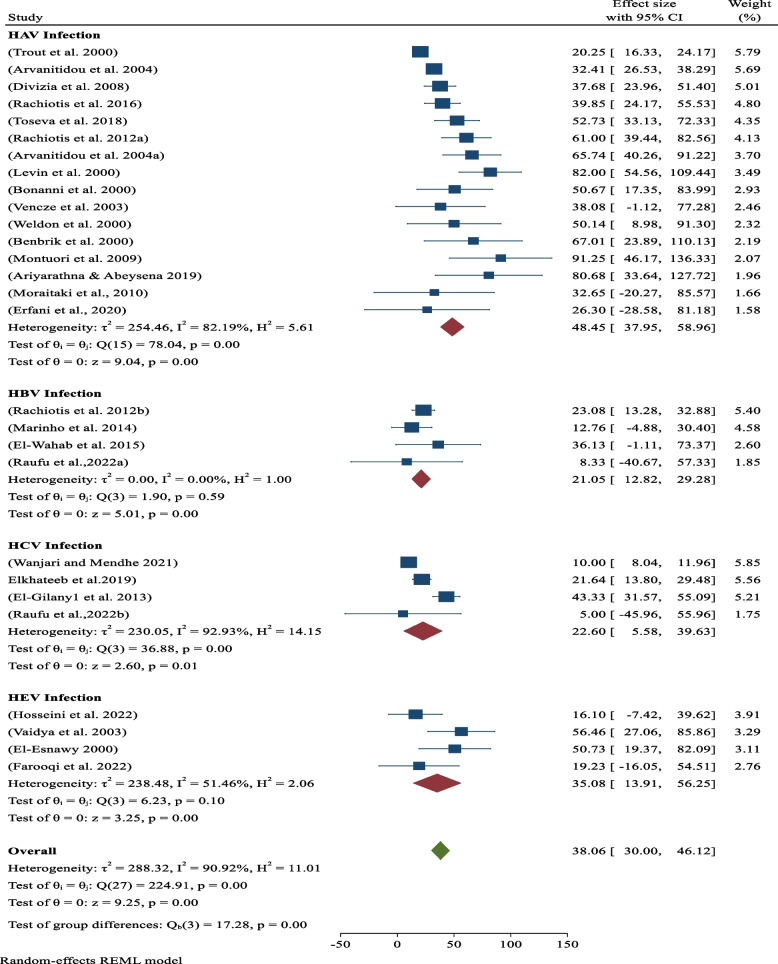
Analysis by Hepatitis viral Type. Sero-prevalence of hepatitis (HAV, HBV, HCV & HEV) among sanitary workers in worldwide

By type hepatitis viral infections sub-analysis, the pooled sero-prevalence of occupational related hepatitis A infection (HAV) among sanitary workers was 48.45% (95% CI: 37.95–58.96; *p*-value < 0.05) in the worldwide and followed by 35.08% (95% CI: 13.91–56.25; *p*-value < 0.05) for pooled prevalence of hepatitis E infection (HEV) (Fig. 4).

### Sero-prevalence of hepatitis year-by-years

**Fig. 5:**
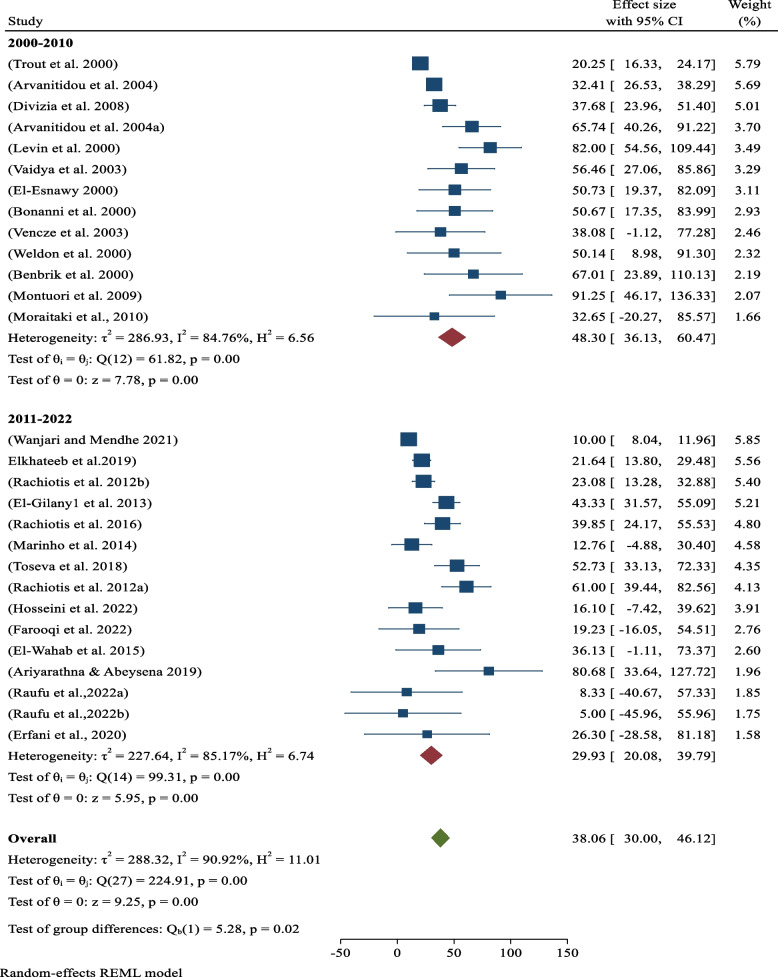
Analysis Year-by-Year. Sero-prevalence of hepatitis by Years among sanitary workers across world

Based on year-by-year sub-analysis, the pooled sero-prevalence of occupational related hepatitis among sanitary workers from 2000 to 2010 years was 48.30% (95% CI: 36.13–60.47; *p*-value < 0.05) and 29.93% (95% CI: 20.08–39.79; *p*-value < 0.05) from 2011 to 2022 (Fig. 5).

### Sensitivity Analysis

**Fig. 6:**
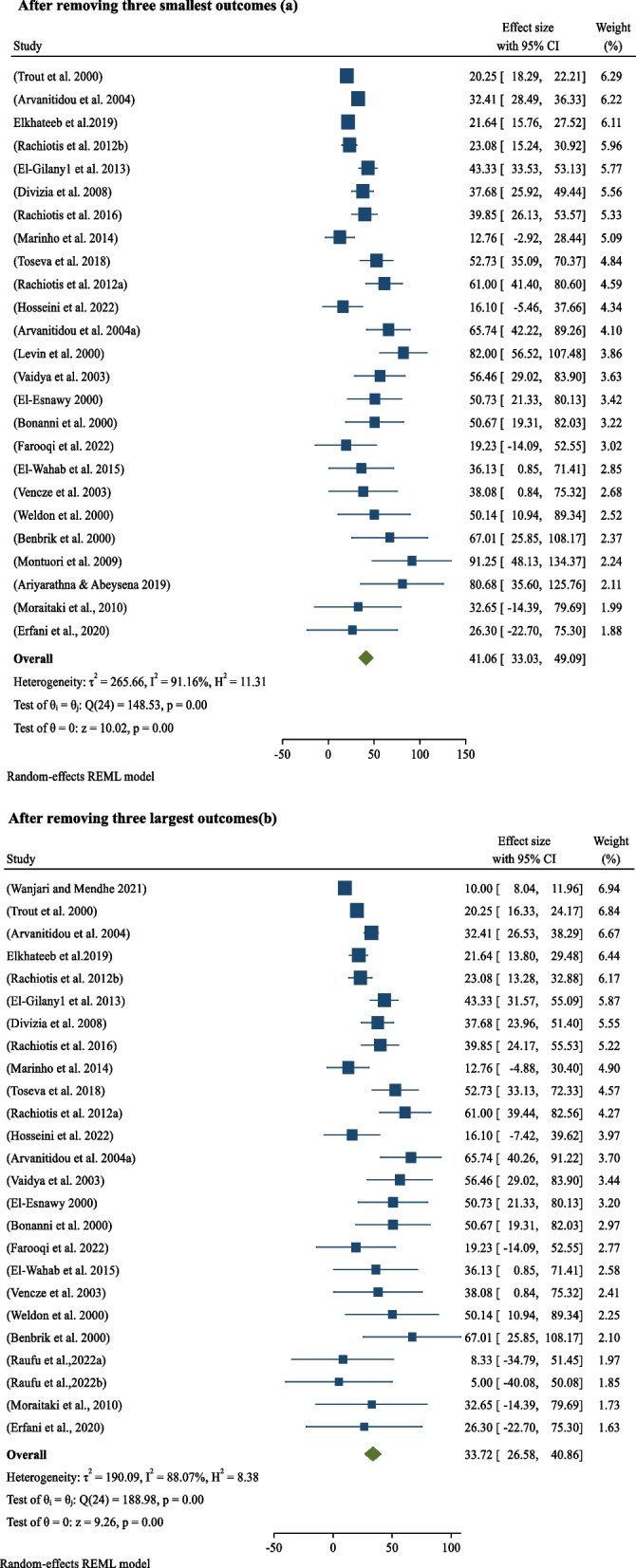
Sensitivity Analysis. After removing three smallest outcomes (**a**). After removing three largest outcomes(**b**). Sensitivity analysis after removing two smallest outcomes(**a**) and three largest outcomes(**b**)

After removing three smallest outcomes (Fig. 6a) and three largest outcomes (Fig. 6b), the previous sero-prevalence of hepatitis viral infections (38.06% (95%CI: 30.00–46.12; *p*-value0.05) among sanitary workers worldwide was found to be 41.06% (95%CI: 33.03–49.09; *p*-value0.05) and 33.72% (95%CI: 26.58–40.86; *p*-value0.05).

### Publication Bias

**Fig. 7:**
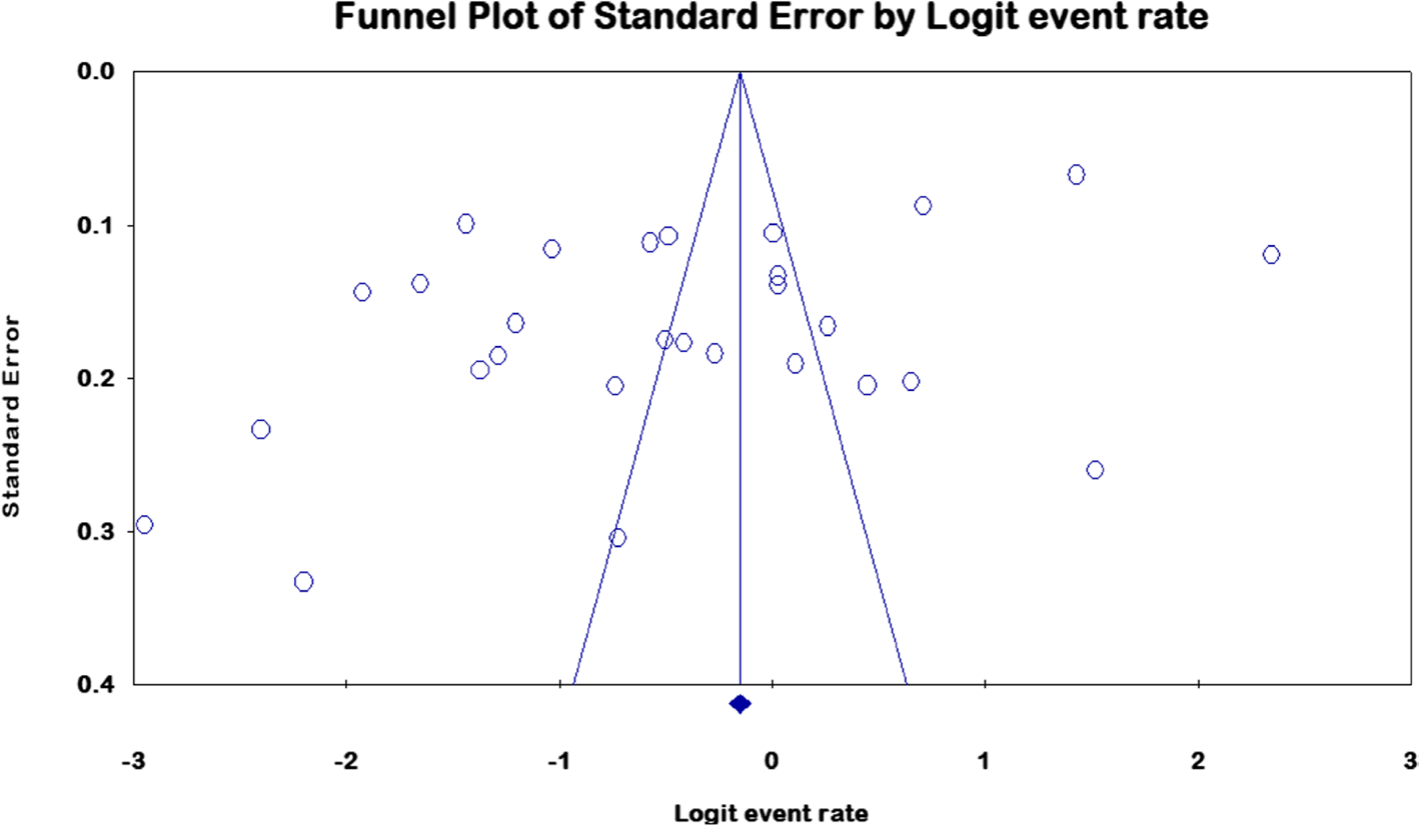
Publication Bias for eligible found from high-income and low-income countries

The JBI criteria for cross-sectional studies, which are comprised of nine assertions, were used to assess the papers' and studies' overall quality was 76.1%, in which the papers met the JBI criteria from two hundred fifty two (28*9)-points (Sup. Table 2). Of these, More over half (56%) have a medium publishing bias, whereas the remainder (44%) have a low one (Table 1). Moreover, statistically, the funnel plot demonstrates that the scatter plots in the image are asymmetrical, with every scatter at *p*-value 0.05 heading away from the funnel's vertical line and center(CI:95%) (Fig. 7).

## Discusion

From the databases, other retrieved data and reports, a total of 182 studies were identified. Of these, One hundred thirty-two studies were from new studies through databases, thirty-eight studies were from new studies through other methods, and twelve studies were from studies included in the previous version of the review. Prior to screening, approximately 65 studies were removed due to record duplication, records marked as ineligible by automation tools, and other factors. Then a total of 115 studies were selected for screening. We excluded 89 studies after screening and of these, 79 studies obtained from the new identification while 10 studies obtained from the previous systematic review. This exclusion was due to a mismatch in study design, publication year, a lack of complete information, and mixed studies with other professions. Finally, the current systematic review and meta-analysis comprised twenty-eight studies to evaluate occupationally associated sero-prevalence of hepatitis viral infectons among sanitary workers across worldwide, in low-income-high-income countries, and within sanitary worker sub-groups. (Fig. 1).

About twelve (*N* = 12) countries across the world were eligible for this systematic review and meta-analysis to assess the pooled prevalence of hepatitis virus infections among sanitary workers. Five were from low-income countries, while seven were from high-income countries (Sup Table 1). The studies were found from Greece [18–22], Italy [23–25], Israel [26], Bulgaria[27], USA[28–30], Brazil [31, 32], SriLanka[33], Egypt [34–38], India [39, 40], Iran[41], Pakistan[42], Nigeria[43] and Iran[44]. Regarding to eligble population, about seven thousand nine hundred forty five sanitary workers were included, and they summarized in supplementary material (Sup. Figure 1). Of these, sixty six percent were of them sewage with waste treatment workers or liquid waste treatment workers, which were reported by sixteen studies [18, 23–31, 33, 34, 38, 39, 42]. The second eligible populations were solid waste collectors those shared twenty five percent were found from nine studies [20–22, 32, 35–37, 40, 43] across worldwide. While, the three studies [19, 41, 44] were conducted on street sweepers which was accounted nine percent from a total sanaitry workers (Sup. Figure 1). In terms of study design, nearly all of the studies used a cross-sectional study design, with sixteen studies using clinical examinations or bio-samples (blood test or serological analysis). Six studies used standard questionnaires as the sole tool for assessing hepatitis viral infections, while two studies used machine detectors of hepatitis viral infections (HEV and HBV). Only one study used serological analysis in conjunction with standard questionnaires (Sup. Figure 2).

Globally, the pooled sero-prevalence of occupational-related all hepatitis infections among sanitary workers was 38.06% (95% CI: 30–0.46.12), which is statisitically associated with work condition (*p*-value: 0.05). From this cumulative sero-prevalence of hepatitis viral infections among sanitary workers was 42.96% (95% CI: 32.63–53.29; *p*-value < 0.05) in high-income countries and 29.81% (95% CI: 17.59–42.02; *p*-value < 0.05) found from low-income countries (Fig. 2) in decreasing order respectively. Contrary to popular belief, high-income countries really paid more attention to sanitary workers than low-income ones. As the result, the gap might be caused by sampling error and study methodological bias among the studies obtained from low-income countries. Based on a occupations subgroup analysis, the pooled sero-prevalence of hepatitis viral infections was 47.66% (95%CI: 37.42–57.90; *p*-value < 0.05) among sewage and waste treatment workers or liquid waste management. In addition, the the pooled sero-prevalence of hepatitis viral infections solid waste collectors and street sweepers were 25.89% (95%CI: 13.82–37.96; *p*-value < 0.05) and 19.82% (95%CI: -0.19–39.84; *p*-value < 0.05) for solid waste collectors and street sweepers, respectively (Fig. 3). As result shows above, the present evidence indicated that hepatitis virus infections are more common in sewage and waste treatment workers than in solid waste collectors and street sweepers. This is due to the fact that the hepatitis A virus is usually found in sewage, waste treatment, and liquid wastes. According to epidemiological data, sewage and waste treatment workers are more likely to contract HAV than other sewage and waste treatment workers since sewage/waste water is the virus's primary host [45].

By type hepatitis viral infections sub-analysis, the pooled sero-prevalence of occupational related hepatitis A infection (HAV) among sanitary workers was 48.45% (95% CI: 37.95–58.96; *p*-value < 0.05) in the worldwide, which was highest as compared to the other three type of hepatitis viral infections (HBV, HCV and HEV). The second common type of hepatitis virus infections found among sanitary workers was hepatitis E infection (HEV), that has been found 35.08% (95% CI: 13.91–56.25), statistitically significant at *p*-value of < 0.05) (Fig. 5). The this evidence indicated it mostly obtained from the low-income countries and this might be due to the sanitary workers are exposed to raw untreated sewage samples, which is host for hepatitis virus [34]. The third type of hepatitis viral infection included in this review is hepatitis C virus (HCV). It was 22.60% (95% CI: 5.58,39.63; *p*-value < 0.05) among sanitary workers, which was most prevalent in sanitary workers (Fig. 5). As contrast, it was lower than the other hepatitis A viral infections and hepatitis E viral infections, but higher that hepatitis B virus infections. The fourth type of hepatitis viral infection included in this review is hepatitis B virus, which shared 21.05% (95% CI: 0.12.82–29.28; *p*-value < 0.05) across worldwide. Such type of virus is in healthcare facilities and the exclusion of sanitary workers who are associated to blood contamination from this analysis. Despite this, the current sero-prevalence of hepatitis B virus (19%) is higher ( almost twice higher) as contrast the finding (11%) obtained from the previous systematic review and meta-analysis [11]. The disparity could be attributed to the actual heterogeneity of the studies between the current and earlier systematic reviews and meta-analyses.

Moreover, in this systematic review and meta-analysis, sero-prevalence of occupationally associated hepatitis viral infections among sanitary workers was also assessed to determine the burden between 2000 to 2010 and 2011 to 2022. Accordingly, the pooled sero-prevalence of occupational associated hepatitis among sanitary workers was 48.30% (95% CI: 36.13–60.47; *p*-value0.05) from 2000 to 2010 and 29.93% (95% CI: 20.08–39.79; *p*-value0.05) from 2011 to 2022. (Fig. 5). This demonstrates that the prevalence of hepatitis viral infections decreases over time, which may be due to increased occupational health and safety service awareness by institutions and sanitary workers (such as proper use of personal protective equipment, post-exposure prophylaxis, creating safe work, and training).

The other work activity in this systematic review and meta-analysis was sensitivitiy analysis. Consequently, after removing three extreme smallest hepatitis outcomes, the pooled prevalence of sero-prevalence of hepatitis viral infection among sanitary workers in wordwide was 41.06%(95%CI: 33.03–49.09; *p*-value < 0.05) (Fig. 6a). Similarily, After removing three extreme largest outcomes, the pooled prevalence of sero-prevalence of hepatitis viral infection among sanitary workers in worldwide was 33.72%(95%CI: 26.58–40.86; *p*-value < 0.05)(Fig. 6b). Hence, the findings are indicating that there is a variation between the previous pooled prevalence and after extreme values are removed, which may lead to publication bias.

Furthermore, meta-regression performed to test heterogeneity, identify true effect, variance of the study and mean effect size of the studies. As a result, the heterogeneity I^2^ (I squared) of the studies was 90.91% (91%), which ranges between 75 and 100%. According to Higgins' interpretation, such type of percentage is indicating significant heterogeneity for the unaccounted variability owing to residual heterogeneity in this review [46], On the other hands, this I^2^ value at a *p*-value of < 0.05 indicates that the observed effect variance is due to actual effect variance as opposed to sampling error. In otherwords, it imlies that the the variation in study outcomes between studies have large degree of heterogeneity (large between-variance), because we have more certainty that the differences in the point estimates among the studies. In this meta-analysis, the real value of Tua square was 288.32, demonstrating how much the true effect sizes differed from one another. In addition, the Q-statistic with 28 degrees of freedom and a *p*-value of 0.05 for heterogeneity in the true effect size or test of homogeneity was 224.91 (Fig. 2). The Q-statistic provides a test of the null hypothesis that all studies in the analysis share a common effect size. Using a criterion alpha of 0.100, we can reject the null hypothesis that the true effect size is the same in all these studies. Therefore, the true effect size in 95% of all comparable populations falls in this interval. Besides, he the mean effect size was conserved where the obtained value is 0.371 with a 95% confidence interval of 0.272 to 0.482 (Sup.Fig. 3), that indicated the mean effect size in the universe of comparable studies could fall anywhere in this interval. In contrast, if the mean effect size is less than 0.500, Cohen's interpretation implies that the variance of the research has a medium impact size rather than a little (0.2) or big (0.8) effect size [47], as shown in this report.

Moreover, the scatter dots on the funnel plot's statistical evidence are far distant from one another, distributed, and away from the central funnel's vertical line. We can estimate the prediction interval as 0.043 to 0.886 if we assume the true effects are normally distributed (in logit event rate). In 95% of all comparable populations, the true effect size falls within this range (Fig. 7). This suggests that the selection bias that is a major issue in this analysis is caused by the presence of bias resulting from chance and the poor methodological quality of smaller studies, where selection bias is very predominant problem in this review. The result found from the critical appraisal assessment also revealed selection bias since it failed to properly sample study participants, address the target population with the sample frame, or apply reliable methodologies to determine the condition. Most studies didn't describe the inclusion/exclusion criteria and method of selection for the workers who handle workplace cleanliness (Sup.Table 2).

## Strengthen and limitations

### Strengthens

Many of the eligible studies had the correct study design, total population, and size, making it straightforward for us to import the data into the programs and complete our objectives within the time frame. Furthermore, investigations on sero-prevalences among various types of sanitary workers were characterized in such a way that it was clear that it was caused by occupationally associated hepatitis virus infections, resulting in a simple search strategy.

### Limitations

There was little study on hepatitis viral infections in review and meta-analysis, notably from low-income countries compared to high-income ones, which may lead to unequally distributed studies around the world. As a result of gaps in research and scientific rigor, the extent to which existing research may serve as an acceptable basis for policy or even estimates of illness burden is severely limited. Furthermore, the current study suggested that studies were confined to cross-sectional studies, implying that future research should focus on longitudinal studies among sanitary workers. As a result, it is envisaged that research will address any gaps in the future, particularly in low-income nations where methodological approaches may induce bias.

## Conclusion

The consistency of the present evidence implies that sanitary workers, particularly sewage workers, are prone to occupationally associated hepatitis viral infection, including hepatitis A, B, C, and E viruses. For further, to conduct a risk assessment of hepatitis viral infections among sanitary worker, a skilled occupational health and safety professional should be engaged. To reduce hepatitis risks among sanitary workers, all sanitary workers, particularly those who handle sewage, should receive disease prevention training, and hepatitis viral vaccination for sanitary workers exposed to sewage should be made available as a matter of necessity in collaboration with local health authorities. In addition, drastic changes in occupational health and safety practices, norms, and instructions, as well as amendments to national and worldwide government laws, are required. In terms of future research, the current review found that there were limited studies on hepatitis virus infections among sanitary workers in low-income countries, underlining the need for future research in these areas that includes longitudinal studies.

## Supplementary Information

**Additional file 1:** **Sup. Table 1.** Eligible countries and number ofidentified studies.** Sup. Table 2.** Overallstudies result by nine statement of JBI.**Sup. Figure 1.** Categories of sanitary workersexposed to HVIreviewed 2000-2022.** Sup. Figure 2.**Tool assessment used.** Sup. Figure 3.**Distribution of True Effect.

## Data Availability

The datasets used and analysed during the current review are available from the corresponding author on reasonable request.
