# Supplementary material for: Sero-prevalence of hepatitis viral infections among sanitary workers across worldwide: a systematic review and meta-analysis
Source: BMC Infect Dis. 2023 Jun 13;23:404. doi: 10.1186/s12879-023-08354-1 (PMC10265919; doi:10.1186/s12879-023-08354-1)
Supplement: Supplementary file 1 — Additional file 1: Sup. Table 1. Eligible countries and number ofidentified studies. Sup. Table 2. Overallstudies result by nine statement of JBI.Sup. Figure 1. Categories of sanitary workersexposed to HVIreviewed 2000-2022. Sup. Figure 2.Tool assessment used. Sup. Figure 3.Distribution of True Effect. [file 12879_2023_8354_MOESM1_ESM.docx]

**Supplemntary Material**

Sup. Table 1 Eligible countries and number of identified studies

| **s.no** | **Name of Countries** | **Categories** | **No of studies Found** |
| --- | --- | --- | --- |
| 1 | Greece | Developed | 6 |
| 2 | Italy | Developed | 3 |
| 3 | Israel | Developed | 1 |
| 4 | India | Developed | 2 |
| 5 | Iran | Developed | 2 |
| 6 | Egypt | Developing | 5 |
| 7 | USA | Developed | 3 |
| 8 | Brazil | Developed | 2 |
| 9 | Sri Lanka | Developing | 1 |
| 10 | Pakistan | Developing | 1 |
| 11 | Bulgaria | Developing | 1 |
| 12 | Nigeria | Developing | 1 |
|  |  | - Developed Countries = 7 - Developing countries= 5 | Total studies=28 |

Sup. Table 2 Overall studies result by nine statement of JBI

| **Statement of JBI for Identified Studies (n=28)** | **Total Yes/28)** | **%** |
| --- | --- | --- |
| 1. Was the sample frame appropriate to address the target population? | 19 | 67.9 |
| 1. Were study participants sampled in an appropriate way? | 13 | 46.7 |
| 1. Was the sample size adequate? | 24 | 86.7 |
| 1. Were the study subjects and the setting described in detail? | 24 | 84.4 |
| 1. Was the data analysis conducted with sufficient coverage of the identified sample? | 21 | 75.6 |
| 1. Were valid methods used for the identification of the condition? | 20 | 71.1 |
| 1. Was the condition measured in a standard, reliable way for all participants? | 23 | 81.1 |
| 1. Was there appropriate statistical analysis? | 24 | 86.7 |
| 1. Was the response rate adequate, and if not, was the low response rate managed appropriately? | 26 | 94.4 |
| **Overall evaluation** | 194 | 76.1% |

**Number of Eligible Population**

Sup. Figure 1 Categories of sanitary workers exposed to HVI reviewed 2000-2022

Sup. Figure 2 Tool assessment used

Sup. Figure 3: Distribution of True Effect
